# Expression of polyglutamine repeats at the pathogenic threshold modestly enhances tau neurotoxicity and protein accumulation in *C. elegans*

**DOI:** 10.17912/micropub.biology.001667

**Published:** 2025-07-31

**Authors:** Joshua C. Hincks, Jade G. Stair, Nicole F Liachko

**Affiliations:** 1 Geriatric Research Education and Clinical Center, VA Puget Sound Health Care System, Seattle, Washington, United States; 2 Division of Gerontology and Geriatric Medicine, Department of Medicine, University of Washington, Seattle, WA 98104, USA

## Abstract

CAG repeat expansions within the HTT gene cause Huntington's disease (HD), a devastating neurodegenerative disease characterized by progressive movement, cognitive, and behavioral symptoms. These expansions result in the expression and accumulation of neurotoxic poly-glutamine (polyQ). Disease initiation depends on the length of the expansion, with fewer than 35 repeats of polyQ typically not pathogenic, while 40 or greater repeats almost always result in HD. Longer expansions correlate with earlier onset of disease; however, there may be other factors that contribute to disease initiation or progression, particularly in individuals with repeat lengths close to or below the 40 repeat length pathogenic threshold. Aggregates of the protein tau are a frequent co-pathology in HD and may modify disease presentation. To examine relationships between tau and polyQ *in vivo* , we generated *C. elegans* co-expressing 40 repeats of polyQ (polyQ(40)) and human tau pan-neuronally. We found that co-expression of tau and polyQ(40) results in mild worsening of motility defects and increased accumulation of total and phosphorylated tau but not polyQ. These results suggest that co-morbid tau and polyQ can worsen neuronal dysfunction, and the presence of tau pathology may contribute to disease phenotypes in patients with HD, particularly individuals with repeat lengths close to the pathogenic threshold.

**Figure 1. PolyQ(40) expression modestly worsens tau motility and increases tau protein accumulation:**
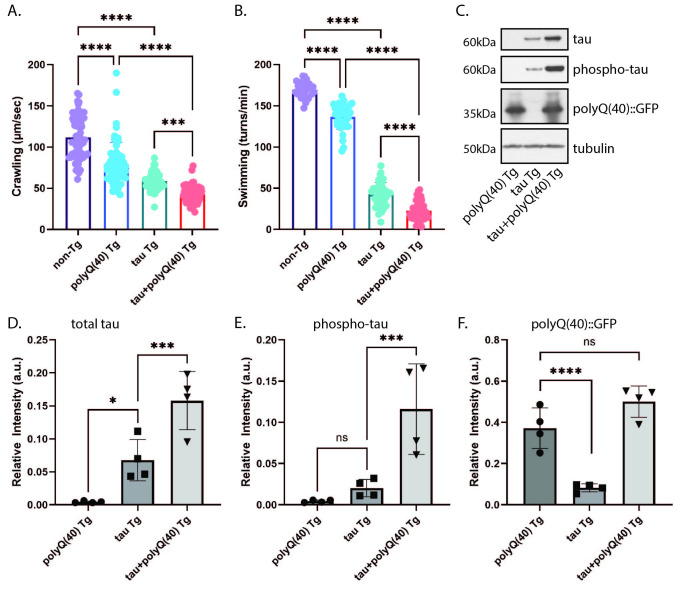
Unstimulated crawling on a seeded agar plate were detected using computer-assisted video tracking and analysis. Movement (center point speed) was recorded (µm/sec). ***p<0.001, ****p<0.0001. N= 240 (non-Tg), 201 (polyQ(40) Tg), 152 (tau Tg), 122 (tau+polyQ(40) Tg), from 4 independent replicates. B) Swimming rates were measured using computer-assisted tracking and analysis. The number of turns per minute were recorded (turns/ min). ****p<0.0001. N= 107 (non-TG), 113 (polyQ(40) Tg), 140 (tau Tg), 127 (tau+polyQ(40) Tg), from 4 independent replicates. C) Developmentally synchronized day 1 adult *C. elegans* were harvested and tested by immunoblot for total tau, phosphorylated tau (AT8), green fluorescent protein (GFP) detecting polyQ(40)::GFP, and tubulin (load control). Immunoblot shown is representative of four independent replicate experiments. D-F) Quantification of protein levels normalized to tubulin. D) Total tau and E) phosphorylated tau are elevated in tau+polyQ(40) Tg animals, while F) levels of polyQ(40)::GFP are unchanged. ns = not significant, *p < 0.05, ***p<0.001, ****p<0.0001.

## Description

Huntington's disease (HD) is a genetically driven neurodegenerative disease characterized by expansions of a CAG repeat within the HTT gene, which lead to pathogenic expression and accumulation of poly-glutamine (polyQ). The CAG repeat length impacts initiation of disease, with 4-26 repeats considered non-pathogenic, 27-35 repeats are an intermediate length at risk of further expansion, 36-39 repeats are likely to cause disease but have reduced penetrance, and ≥ 40 repeats almost always result in disease (Jiang, Handley et al. 2023). Most patients develop HD in early mid-life, between ages 35-40; however, longer repeat lengths lead to an earlier age of onset for patients. Mid-life onset patients typically have repeat lengths ≤50, while most juvenile onset cases of HD have expanded tracts with >60 repeats. It is possible that additional pathological factors contribute to development or presentation of HD, particularly in patients with intermediate repeat lengths close to the pathogenic transition point. Aggregates of the protein tau comprise the major causative neuropathology present in patients with frontotemporal lobar degeneration (FTLD-tau), and contribute to numerous other neurodegenerative diseases including Alzheimer's disease (Lane-Donovan and Boxer 2024). In HD, somewhere between 13-48% of patients exhibit tau aggregates as a co-pathology, and pathological tau is thought to contribute to cognitive decline in these patients (Baskota, Lopez et al. 2019, Salem and Cicchetti 2023).

We have previously found that *C. elegans* models co-expressing human tau (tau Tg) and strongly pathogenic 86 repeats of polyQ (polyQ(86) Tg) do not exhibit worsened movement or exacerbated protein accumulation, excluding either additive or synergistic neurotoxicity between these two proteins (Latimer, Stair et al. 2022). It is possible that tau and polyQ have overlapping mechanisms controlling their neurotoxicity, which are already maximally affected in the severely impaired polyQ(86) expressing animals and thus no further enhancement is possible. If there are shared neurotoxic pathways between tau and polyQ, a less severe model of polyQ neurotoxicity could uncover this relationship. To test this possibility, we utilized *C. elegans* expressing 40 repeats of polyQ tagged with GFP (polyQ(40) Tg), which have milder neuronal dysfunction than polyQ(86) Tg (Brignull, Moore et al. 2006, Brignull, Morley et al. 2006, Gidalevitz, Ben-Zvi et al. 2006). We crossed tau Tg and polyQ(40) Tg animals to generate double transgenic animals homozygous for both tau and polyQ(40) (tau+polyQ(40) Tg), which express both human tau and polyQ(40) pan-neuronally. We then assessed locomotor behavior, which serves as a sensitive readout of neuronal function. Both tau Tg and polyQ(40) Tg animals exhibit defects in unstimulated crawling and swimming motilities on their own ( Figure 1A- B). We found co-expression of tau and polyQ(40) modestly enhanced these defects, with significantly greater impairment in both crawling speed and swimming rate than the single-transgenic strains.

To examine whether these behavioral impairments were associated with changes in protein levels, we performed western blot analysis on lysates from staged day 1 adult animals. Western blot analysis demonstrated that both total and phosphorylated tau protein levels were elevated in tau+polyQ(40) Tg animals relative to those expressing tau alone ( Figure 1C- E). PolyQ(40) levels were detected using a GFP antibody; interestingly there were no significant differences in levels of polyQ(40) between the single and double transgenic animals ( Figure 1C, F). However, previous work found that strongly pathogenic polyQ(86) did not lead to increased total or phosphorylated tau protein in *C. elegans* (Latimer, Stair et al. 2022), suggesting the length of the polyglutamate chain determines its impact on the accumulation on tau *.* Although additional work will be needed to examine the mechanisms controlling this relationship, these data support a relationship between polyQ and tau where the presence of moderate polyQ repeat lengths can promote tau accumulation, leading to worsened neuronal dysfunction, potentially through enhancement of a shared pathogenic pathway.

A number of important questions can be pursued using this new model of tau and polyQ co-expression. These include whether tau or polyQ influence each other's aggregation, localization, or spreading throughout the nervous system. While we did not observe qualitative changes in polyQ(40)::YFP puncta in the presence of tau, a more in depth analysis may detect differences. Use of a fluorescently tagged tau could also be used to allow simultaneous imaging and analysis of tau and polyQ(40) expression and localization in living neurons. Fluorescently tagged proteins or immunofluorescent immunostaining could be used to detect whether tau and polyQ(40) co-localize. Prior work with tau transgenic strains indicate that tau is predominantly present diffusely throughout the cytoplasm of the cell body (Kraemer, Zhang et al. 2003, Han, Saxton et al. 2024). Given the similar diffusely cytoplasmic localization of polyQ(40) (Brignull, Moore et al. 2006), we believe it is likely that they generally co-localize. Sequential extraction of proteins with detergents of increasing solubilizing strengths could also be used to detect solubility changes indicative of aggregation by Western blot (Ishihara, Hong et al. 1999). Finally, expression of fluorescently tagged tau or polyQ(40) in a subset of neurons could allow visualization and enhancement of cell to cell spreading, which is a likely contributor to disease progression in tauopathies.

## Methods

*C. elegans* husbandry and strain generation

Strains were maintained as previously described (Brenner 1974). NLS48 was generated by crossing AM101 with CK1441 to generate a double transgenic animal expressing both human tau and poly-Q40. All strains are generated and tested as homozygotes for their various transgenes.

Motility assays and analysis

Unstimulated activity (crawling) and swimming (thrashing) behaviors were assessed using the WormLab system (MBF Bioscience) (Currey and Liachko 2021). For crawling assays, stage-matched day 1 adult *C. elegans* were transferred to 35 mm NGM assay plates seeded with 20 μl OP50 bacteria. Animals were allowed to acclimate to conditions on assay plates at room temperature for at least 30 min before recording movements for 1 min at 7.5 frames per second. For thrashing assays, staged-matched day 1 adult *C. elegans* were given 30 min to acclimate to the assay room conditions. Approximately 50 animals were then transferred to a 35 mm assay plate by way of washing in 1 ml M9 buffer, given 1 min to standardize swimming behavior, followed by a 1 min recording time at 14 frames per second. Tracks were verified and repaired as needed. Figures show results from at least three independent replicates.

Immunoblotting and quantitation

Approximately 10,000 stage-matched day 1 adult *C. elegans* were harvested and snap frozen per sample. Protein was extracted by resuspending pellets in 1× sample buffer, three sessions of 10 seconds sonication with cooling on ice water in between sessions, and 10 min boiling. Samples were loaded and resolved on precast 4-15% gradient SDS-PAGE gels and transferred to PVDF membrane as recommended by the manufacturer (Bio-Rad). Chemiluminescent signals were detected on film, capturing a range of signal intensities. Quantitation was completed by ImageJ software densitometry analysis of scanned film images chosen within the linear range of signal detection.

Statistical analyses

All statistical analyses were performed using GraphPad Prism statistical software. Statistical significance was determined using one-way ANOVA with Tukey's multiple-comparison test. Error bars represent standard deviation.

## Reagents

Strains

N2 (Bristol).

AM101 *rmIs110 [F25B3.3p::Q40::YFP]* [6]

CK1441 *bkIs1441 [Paex-3::Tau WT(4R1N)+Pmyo-2::dsRED]* [9]

NLS48 *rmIs110 [F25B3.3p::Q40::YFP]; bkIs1441 [Paex-3::Tau WT(4R1N)+Pmyo-2::dsRED]*

Antibodies used for immunoblotting

Human tau: anti-tau (A0024, Dako, 1:200,000).

Phosphorylated tau: anti-phosphorylated tau (AT8/PHF-Tau, MN1020, Thermo Fisher Scientific, 1:1000)

β-tubulin: antibody E7 (Developmental Studies Hybridoma Bank, 1:5000).

Secondary antibody: HRP-labeled goat anti- mouse IgG (Jackson ImmunoResearch, 115-005-003, 1:2500). HRP-labeled mouse anti-rabbit IgG secondary antibody (Jackson ImmunoResearch, 211-005-109) was used at a dilution of 1:10,000.
